# Selective capture and digital counting of intact HIV pseudovirus using designer DNA nets, tethered motion, and photonic resonator interferometric scattering microscopy

**DOI:** 10.1063/5.0289982

**Published:** 2026-04-01

**Authors:** Joseph Tibbs, Saurabh Umrao, Tingjie Song, Varada Anirudhan, Skye Shepherd, Katy Wolhaupter, Mengxi Zheng, Sydney Wiggins, Lijun Rong, Colin L. Hisey, Utkan Demirci, Xing Wang, Brian T. Cunningham

**Affiliations:** 1 Department of Bioengineering, University of Illinois Urbana-Champaign, Urbana, Illinois 61801, USA; 2 Nick Holonyak Jr. Micro and Nanotechnology Laboratory (HMNTL), University of Illinois Urbana-Champaign, Urbana, Illinois 61801, USA; 3 Carl R. Woese Institute for Genomic Biology (IGB), Urbana, University of Illinois Urbana-Champaign, Urbana, Illinois 61801, USA; 4 Department of Chemistry, University of Illinois Urbana-Champaign, Urbana, Illinois 61801, USA; 5 Department of Microbiology and Immunology, University of Illinois Chicago, Chicago, Illinois 60612, USA; 6 Department of Biomedical Engineering, Northwestern University, Evanston, Illinois 60208, USA; 7 Bio-Acoustic MEMS in Medicine (BAMM) Lab, Canary Center at Stanford for Cancer Early Detection, Stanford School of Medicine, Stanford University, Palo Alto, California 94304, USA; 8 Department of Electrical and Computer Engineering, University of Illinois Urbana-Champaign, Urbana, Illinois 61801, USA; 9 Cancer Center at Illinois, University of Illinois Urbana-Champaign, Urbana, Illinois 61801, USA

## Abstract

Rapid and quantitatively accurate detection of HIV (human immunodeficiency virus) viral load using a simple workflow, automated instrumentation, and real-time data processing with easily interpretable output is required for an approach to become practical for point-of-care environments. We recently demonstrated a form of interferometric scattering microscopy called Photonic Resonator Interferometric Scattering Microscopy (PRISM) that amplifies the contrast of surface-attached nano-objects via a photonic crystal (PC) surface. Recently, our team also developed net-shaped DNA nanostructures called “Designer DNA Nets” (DDN) that organize multivalent aptamer arrays to precisely match the pattern of proteins on the outer surface of intact virions to provide high-affinity and selective binding. In this work, we demonstrate the combination of DDNs and PRISM for detection of HIV by digital counting of captured viruses. We compare multivalent DDN-based viral capture to monomeric aptamer and nanobody capture, in which the captured virions are tethered to the PC surface by a DNA linker. We observe that tethered virions are not fully stationary and that their localized dynamic movement provides a route for label-free digital-resolution detection with a signal-to-noise ratio of 50, while disregarding the presence of image features not related to specific virus capture. We obtain a detection limit of 10^4^ virions/ml with a single-step, room temperature 30-min assay and excellent selectivity for non-detection of a nonspecific virus and the presence of a high concentration of extracellular vesicles. This study highlights PRISM's utility as a means for versatile detection of immobilized particles as part of an assay for affinity molecule evaluation.

## INTRODUCTION

Acquired immunodeficiency syndrome (AIDS), caused by infection with human immunodeficiency virus (HIV), continues to impact 39 million people worldwide, and regular monitoring of viral load is an essential tool for reducing morbidity and detecting drug resistance or improper treatment adherence. Access to frequent, accurate, and highly sensitive HIV viral load monitoring is a critical component of early infection diagnosis, HIV antiretroviral therapy, and routine diagnostic testing to keep people informed and help them maintain their HIV viral load status. Despite available treatments, there are many barriers to sensitive testing for HIV outside of specialty clinical settings, leaving many unable to monitor their infection status.^1,2^ In the US, 12.7% of people with HIV (PWH) are unaware of their HIV-1 status.^3^ Rapid, simple, and inexpensive multiplexed detection of viruses such as HIV, HBV, and HCV viral loads in whole blood would enable testing in locations without an onsite lab to improve access to timely diagnostics, offer personalized care, aid in disease surveillance, and promote a patient-centered approach to healthcare delivery.^4–8^ The resulting capability will be most transformative for underserved groups (e.g., unstably housed people, who use drugs, people with HIV, and formerly incarcerated populations) where rates of co-occurring infections are the highest.^9^

**FIG. 1.**
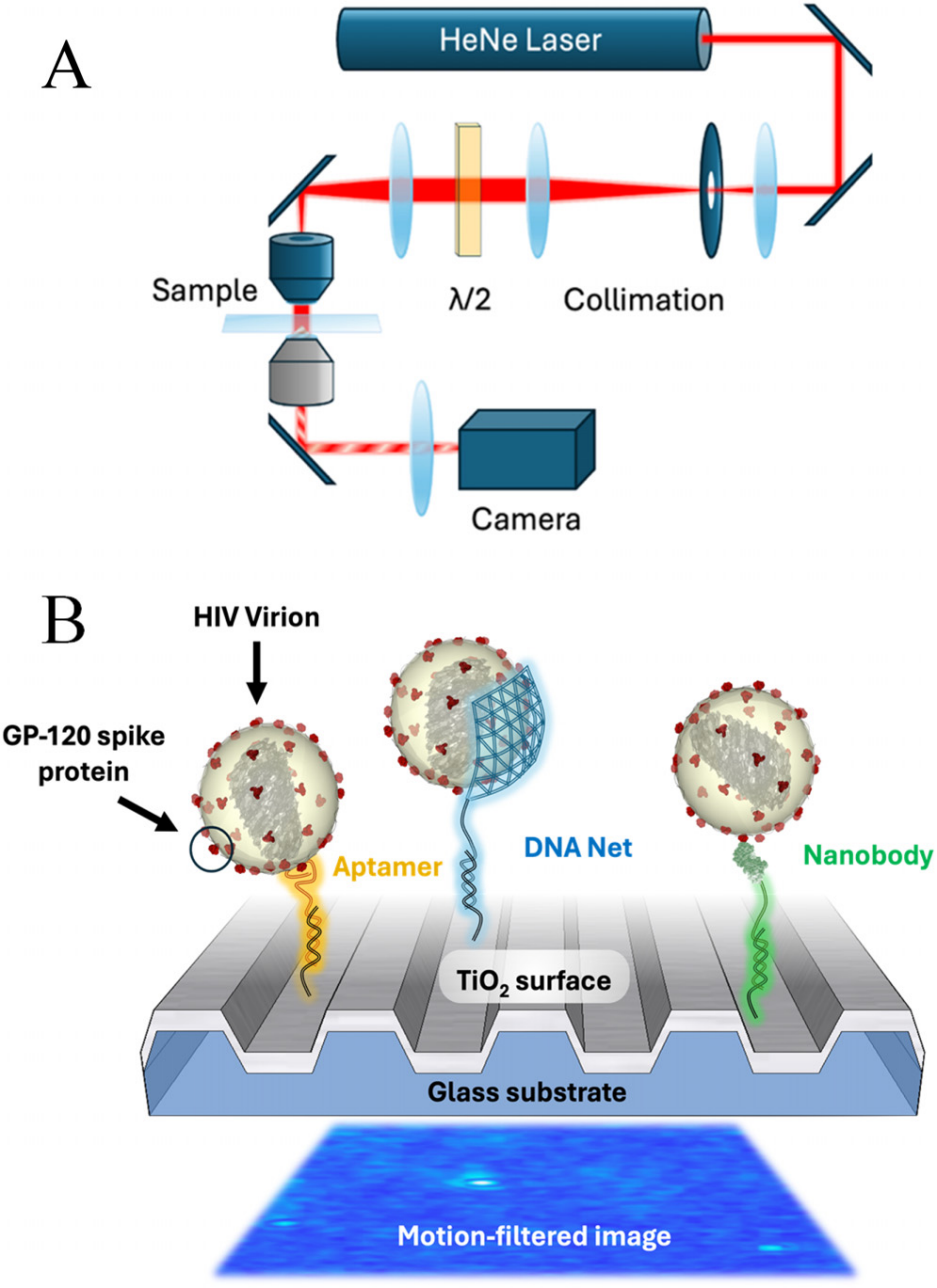
Overview of PRISM. (a) Schematic view of the PRISM system components. Light from a helium–neon laser is collimated through a 25 *μ*m pinhole before passing through a rotating half-wave plate to align the light's polarization to a TM orientation at the PC biosensor surface. A lens focuses the light on the back focal plane of the illumination objective, resulting in Köhler illumination at the sensing plane. A mixture of attenuated reference beams and scattered photons from particles on the photonic crystal surface is sent to a 100× illumination objective and a CCD image sensor. (b) Schematic of the three capture strategies shown interacting with HIV virions. From left to right, an aptamer, a DNA net, and a conjugated nanobody are shown tethered using interactions with an ssDNA strand covalently bonded to the surface with silane chemistry. The processed image below shows PRISM data for three captured viruses.

The gold-standard available tests are based on the detection of the viral genome, which has been reviewed extensively in other works, and represent various trade-offs between cost, sensitivity, and point-of-care convenience.^10,11^ Typically, these tests require breaking the viral envelope followed by enzymatic amplification of a specific nucleic acid sequence. As a simpler alternative, several researchers have demonstrated the efficacy of selectively capturing the intact virus upon a biosensing transducer^12–20^ [represented schematically in Fig. 1(a)], or by “decorating” the captured virus with fluorophores or nanoparticle tags that facilitate visualization.^21–25^ The advantage of this style of detection is that the assay is more direct, requires little (if any) sample pre-processing, is more stringent against interfering materials (e.g., enzyme inhibitors), obviates the need for temperature control, and provides simple quantitative output. When intact virions are selectively captured upon the active area of a biosensing transducer, a detectable signal is registered by the accumulated effects of the virus mass (for acoustic biosensors),^26,27^ dielectric permittivity (for optical biosensors),^28–30^ or impedance.^31^ In order for the detected signal to be recognized above background noise, typically many viruses must accumulate on the sensor, and a detection event is therefore the aggregate effect of many viruses.

Interferometric Scattering Microscopy (iSCAT) methods have proven versatile for the label-free detection of single biological particles that interact with a surface within the focal plane. Contrast in iSCAT originates from the optical interference between the electromagnetic field that results from scattering by the particles of interest and the wave that reaches the image sensor from the illuminating reference beam. By normalizing the detected intensity by the reference beam intensity, the cross term of photon interaction that is dependent on phase is extracted.^32–34^ This arrangement leverages the favorable scaling of the scattering cross section, scaled by the third power of the particle's diameter, resulting in signal contrast that is proportional to the particle mass. iSCAT and Mass Photometry (MP) are gaining broad utilization for studies that measure biomolecular dynamics in a mass-sensitive and label-free manner.^33–39^ The most common method of reference beam filtering is to gather continuous videos of a field of view (FOV), from which one can calculate a pixel-wise time average or median value, followed by pixel-wise subtraction of these “stationary” signals to isolate the contributions of transient particles that bind, unbind, or diffuse.^36,40,41^ Rolling-window techniques are used to improve the temporal resolution to examine changes that occur over the timescale of a few frames, allowing observation of multiple binding events in the same location or fast cell dynamics.^42,43^ Effectively, rather than applying one reference beam measurement to all subsequent frames, smaller intervals of time are individually analyzed to determine the background signal for the nearest N frames, and that signal is subtracted from the frame in the middle of that range.^39^ Finally, systematic modulation of the sample can enable lock-in mechanisms to discern particular frequencies of movement,^44^ or a temporal modulation of the light source.^45^ There are also iSCAT image processing approaches for observing unlabeled particles at the end point of a reaction in which the detected nano-objects attach to capture molecules during an incubation step, in which determining the total density of binding is more important than the kinetics of binding events.^35^

Recently, we introduced Photonic Resonator Interferometric Scattering Microscopy (PRISM) as a form of iSCAT that is capable of identifying and counting surface-captured nano-objects on a photonic crystal (PC) surface.^46^ Like iSCAT, PRISM is a label-free detection method in which destructive interference between an illuminating laser and the forward-scattered light from a nano-object (such as a virus) in the focus plane generates contrast at the plane of an image sensor where the illuminating and scattering electromagnetic field components interfere. As discussed in our previous work, the PC enhances the contrast by back-reflecting a large percentage of the illuminating light to reduce the intensity of the reference beam, while the resonant coupling of the illumination with the PC surface provides an enhanced near field that amplifies the forward scattering magnitude from the virus. Furthermore, the PC's dispersion efficiently directs outcoupled scattered photons toward the range of angles captured by the objective lens's numerical aperture. When using PRISM to detect intact virions, any captured virus in the imaging field of view (FOV) can be individually visualized and counted.^12,13^ Unlike conventional biosensing transducers that have only a small active sensing region, the entire PC surface serves as an active sensing area. Our PRISM instrument utilizes a high frame rate image sensor (∼360 frames/s, over a 1.5 s acquisition) and image processing strategies that compare the current frame to the previous frame (or the average of several previous frames) to highlight only subtle *changes* that occurred between the two frame times. In iSCAT and PRISM, the contrast induced by surface attachment of nano-objects is very subtle, and high frame rate imaging with subtraction of very recently obtained (and averaged) images is necessary to remove image noise caused by mechanical vibration, laser intensity fluctuation, or any other common-mode variability in spatial uniformity that occurs at timescales greater than milliseconds. Using conventional boxcar averaging for PRISM image processing, a newly arriving virus that is captured on the surface will briefly appear as a diffraction-limited set of pixels that represent the point spread function (PSF) that are darker than the surrounding pixels where no change occurred. After the boxcar averaging time period advances past the arrival time of the virus, the virus's contribution to scattering is averaged away and becomes part of the background upon which any future arriving virus would be compared against. In this way, a captured virus does not persist in a boxcar-averaged image sequence. Likewise, when a virus comes into contact with the PC surface but is not captured, it may be observed to leave the surface (by diffusion) or to translate laterally across the surface. Using boxcar averaging, a virus that leaves the PC surface appears briefly as a point spread function sized set of pixels with *negative* contrast (when compared to the positive contrast of an arriving virus). A virus that translates laterally will appear to arrive at one set of pixels at its leading edge while appearing to leave a set of pixels at its trailing edge and will appear as a two-sided object with positive contrast at the leading edge and negative contrast at the trailing edge.^40^

While analyzing PRISM image sequences during virus capture, we observed that captured viruses are not stationary, but rather “flicker” while they are tethered to one location. Therefore, while a captured stationary object (such as a dust particle) in a PRISM image sequence will briefly appear and then disappear from a differential image sequence obtained by boxcar averaging, a virus that wiggles provides a persistent image feature that is very easily recognized with a high signal-to-noise ratio. By using a camera with a sufficiently rapid shutter speed, each frame captures the particles' signals at particular positions within their respective constrained diffusion ranges rather than an averaged signal over the full range. Hence, from frame-to-frame, each particle's scattered photons change sufficiently to be detected by image processing methods adapted from established iSCAT principles. Likewise, any nonspecifically attached nano-objects that are similar in size to our targeted virion but that simply land on the sensor by gravity or some other nonspecific attachment mechanism will lack the tether of the specific capture mechanism and remain stationary. Likewise, virions or other nano-objects that laterally diffuse across the sensor surface or that transiently attach (attach and then leave) will not have persistent spatial fluctuations in a single location. Thus, our hypothesis is that observation and counting of image features that display persistent fluctuation in one location will enable an image processing approach that will only provide contrast for specifically captured tethered intact virions.

In this report, we extended our capability for PRISM-based virus counting in two important ways. First, we developed the capability for increasing our imaging field of view by “PRISM Scanning,” in which up to 100 FOVs are combined to yield a total of 100 000 *μ*m^2^ scanned surface area. PRISM scanning ensures that rare viral capture events are found with greater statistical likelihood, utilizing the test sample with low viral load more efficiently. Because the high frame rate capture occurs for a very brief (1.5 s) time, PRISM scanning can cover the sensing area in less than 15 min and thus maintain fast sample-to-answer times. Second, we developed an image processing approach to recognize dynamic features in the PRISM time-series image stack that specifically identify them as captured virions by their localized fluctuation. This dynamic data collection removes the need for reference frames to be taken for background subtraction and allows more flexibility for multi-area scanning.

The capabilities of PRISM for the direct detection of viral particles offer an attractive alternative to conventional molecular assays for virus-specific nucleic acid sequences or viral antigens. For such an approach to be successful, it is essential for low-concentration viruses to be captured on the sensor with sufficient selectivity so that non-target biological nano-objects such as extracellular vesicles (EVs) and lipoproteins that are present in plasma at orders of magnitude greater concentration do not mask the presence of viruses.^47^ Importantly, because intact virion detection does not incorporate a mechanism for enzymatic amplification [as used in polymerase chain reaction (PCR) and enzyme-linked immunosorbent assays (ELISA)], it is necessary to maintain a high efficiency of virus capture and a high signal-to-noise ratio for clearly observing captured viruses.^48^ The purpose of the present study is twofold. First, we seek to compare the efficiency of alternative ligand capture strategies and, in particular, to evaluate multivalent, aptamer-based, and nanobody-based approaches. Second, we seek to utilize a novel approach for clearly recognizing and counting a novel feature of captured and tethered virions that is specific to the label-free PRISM detection approach, in which tethered viruses display rapid motion that differentiates them from nano-objects that are simply adsorbed to the sensor surface. Here, we conducted the study with relatively simple test samples with target and non-target viruses spiked into buffer at known concentrations. We are currently exploring clinical utility by detecting virus spikes in plasma and by conducting detection of plasma samples with clinically relevant viral loads.

This strategy extends the work pioneered by previously reported techniques, which also capture the intact virus and identify it based on affinity to proteins on the viral envelope by immobilizing virions on a transducer surface.^26,49^ Antibodies against HIV capsid protein gp120 are a common component of lateral flow assays for the detection of HIV,^50^ which served as the recognition element in the device used in a 2013 report by Inci *et al.*, which used plasmonic methods to detect very low concentrations.^15^ However, many of these methods rely on measurement of an aggregate of many virions accumulating on the sensor surface. Capture and digital counting of individual virions provides a route toward reduced limits of detection.^46,51^ Other microscopy platforms have similarly developed affinity molecule screening methods to aid in the rapid search for the most effective capture method.^51^ This approach has clear benefits in responding to emerging viruses and viral variants that may render previous gold-standard capture methods less effective.

The landscape of effective affinity molecules is constantly evolving, and each exhibits unique strengths. For instance, the cost and functional stability during shipping and storage limit the effectiveness of antibodies for point-of-care testing environments. Aptamers are single-stranded sequences of RNA or DNA designed to fold into stable shapes that provide high binding affinity for a specific target. Aptamers have the benefit of being easy to design and test with modern sequencing and nucleic acid synthesis technology, computer modeling, and enrichment processes such as SELEX.^52–54^ Unlike antibodies, aptamers may be produced at a large scale without relying on extensive testing of microbial conditions to ensure proper folding. Their cost-effective and rapid development potential makes them attractive for affinity molecules for novel targets.

Previous reports by our team show that multiple aptamers can be incorporated into designer DNA nanostructures (DDNs) to provide multivalent binding to intact virions with affinity that is substantially greater than a single aptamer. Net-shaped DDNs, referred to here as simply “DNA Nets,” are synthesized in a one-pot reaction using established methods with aptamers that are arranged to spatially align with the periodic patterns of viral envelope surface proteins and binding sites.^55,56^ The resulting DDN architecture provides flexibility and broad coverage, enabling it to bind to numerous proteins present on the surface of its target virus. DDNs have been previously demonstrated for the capture of Dengue and SARS-CoV-2 viruses with a 1000- to 10 000-fold reduction in the concentration needed for viral inhibition compared to aptamers. Importantly, DDNs retain their effectiveness in plasma and saliva.^56,57^ Each DDN rapidly conforms to the virus shape to provide rapid and selective high-affinity multivalent binding to provide either fluorometric^58^ or biosensor-based detection,^59^ with detection limits similar to those achieved by polymerase chain reaction (PCR). DDNs have also been demonstrated for rapid detection of the SARS-CoV-2 Trimeric Spike Protein (TSP) spiked into saliva, urine, and serum via a paper-based microfluidics platform, with a broad dynamic range of 10^3^–10^8^ viral copies/ml.^60^

A further alternative to antibodies for the capture of intact virions is to utilize only the variable domain of the heavy chain (VHH), often referred to as “nanobodies.” Nanobodies have been developed for HIV neutralization^61^ and virion detection.^62^ Nanobodies are produced by expression in bacterial vectors, providing the capability for large-scale production. In this study, gp120-recognizing nanobodies were modified with a single-strand DNA (ssDNA) tether [Fig. 1(b)] to facilitate attachment to the PC surface. In this work, we present the characterization of a DDN for the detection of intact HIV pseudovirus, demonstrating a comparison of the selectivity and sensitivity with intact virion capture by a custom DDN, anti-gp120 heavy-chain antibody fragments, and a gp120-recognizing aptamer.

Overall, the goal of our work is to develop a simple and rapid workflow for the detection of pathogenic viruses that does not require nucleic acid extraction, enzymes, or thermal cycles but yet provides nearly equivalent limits of detection. We are considering the limitations of conventional laboratory methods such as PCR that are difficult to translate to point-of-care settings due to the complexity of the assay and the associated cost of instrumentation.^8^ The cost of the PC biosensor is not expected to serve as an impediment for adoption, as we have demonstrated mass-manufacturable versions of the same device fabricated in plastic roll-to-roll format for disposable labware^63^ as well as in 8-in. diameter glass wafer format.^64^ Similarly, we recently reported a portable version of the PRISM instrument that incorporates cost-effective vibration isolation and autofocus with a component cost of ∼$20 000 USD that we expect could be mass-manufactured for <$5000 USD.^13^ Although the PRISM instrument is complex in its current form, the advantages of the reported virus detection approach are the following: (1) Simplicity of the assay workflow with an incubation that does not require temperature control, thermal cycles, or enzymes. (2) Label-free readout that does not depend upon fluorescent dyes that are subject to photobleaching. (3) Rapid readout through the use of a high frame rate image sensor and image sequence analysis. (4) Digital-resolution counting of high signal-to-background detected features over a large sensor field of view. This work seeks to demonstrate the capabilities of Photonic Resonator Interferometric Scattering Microscopy (PRISM) in combination with an image processing method that provides high signal-to-noise detection in captured intact virions, for which we recently demonstrated the capabilities of a portable instrument that would be suitable for environments such as health clinics.^65^ Evaluating nucleic acid-based DDNs as an alternative to protein-based antibodies has potential benefits such as reducing reliance on cold-chain storage, which is not always available in non-laboratory settings.^66^

## RESULTS AND DISCUSSION

As described previously,^12,46^ PRISM is a label-free method for rapid digital-resolution detection of particles such as viruses. It relies on the resonant properties of PCs to generate a high reflectance at the resonance condition of incident light. This results in an attenuation of the reference beam that improves the contrast of the interferometric scattering signal generated, and small particles such as virions can be counted directly on the functionalized surface. The schematic of the optical path of the instrument, which can be built on a commercial microscope base or constructed using off-the-shelf parts,^13^ is shown in Fig. 1(a). In this work, we used PRISM to image HIV pseudoviruses captured by three molecules: anti-gp120 aptamers, DNA nets decorated with anti-gp120 aptamers, and anti-gp120 “nanobodies” [Fig. 1(b)].

**FIG. 2.**
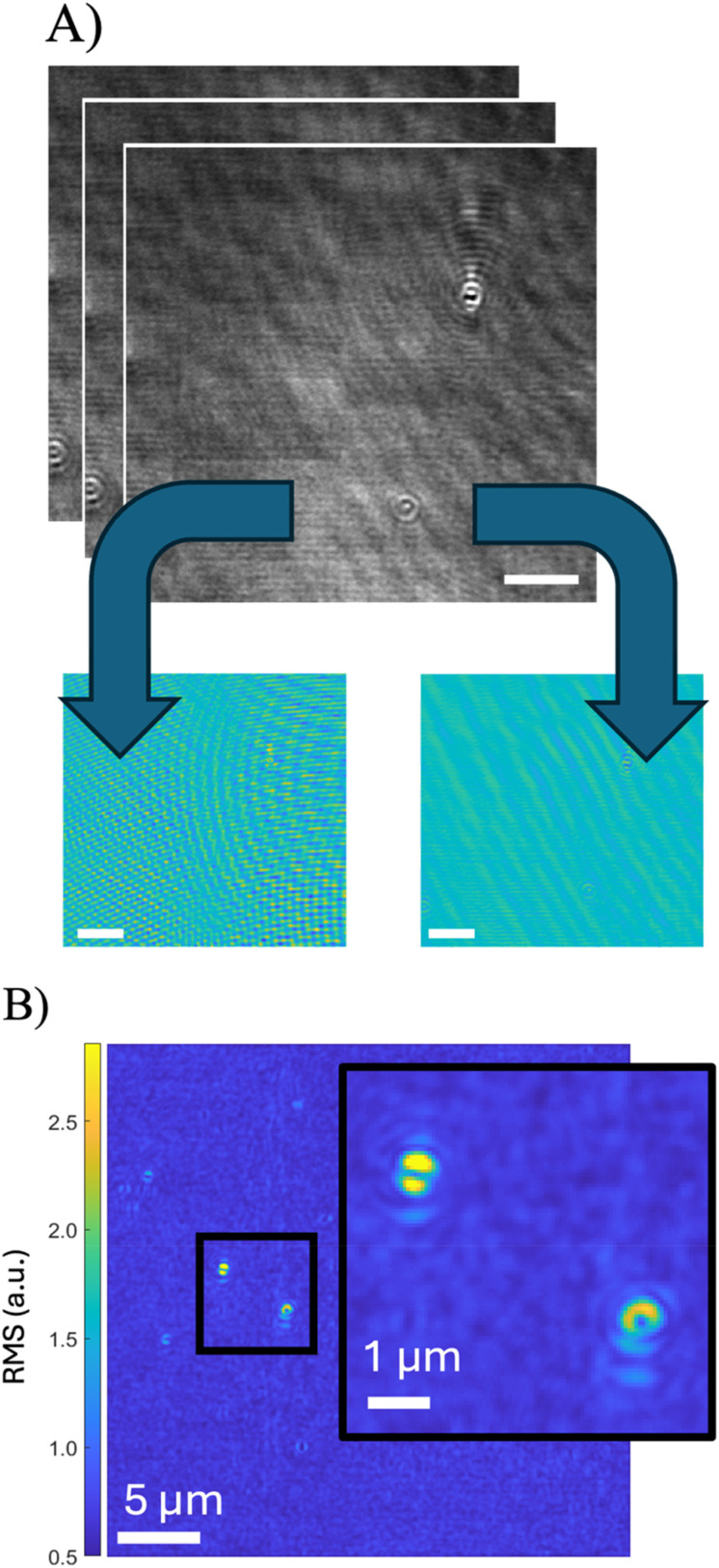
Image processing workflow. (a) The stack of raw images obtained from the camera is subjected to framewise principal component analysis (PCA) to determine sources of noise that systematically affect the entire frame, such as laser fluctuations and background movement. See equations in the supplementary material for a mathematical description. These contributions (inset) are subtracted from the image set. All scale bars are 5 *μ*m. (b) The RMS value of each pixel's frame-to-frame difference is calculated; this identifies localized regions of higher fluctuation independent of the background noise [see Eq. (1)]. The images created by this transformation of the video data clearly show the location of bound particles (inset).

The strategy of incubation followed by imaging increases the potential throughput of the assay, since binding events do not need to be observed as they happen but can be seen after the fact. In order to detect captured intact virions, a modification to conventional iSCAT boxcar averaging image processing was employed. For particles the size of an HIV virus, diffusion during the collection period of the camera (0.05 ms) is relatively unconstrained, as the root mean squared diffusion distance is less than the length of the DNA tether. Therefore, the PSF of the particle in the imaging plane is characteristic of the scattered photons for only a small portion of the particle's total phase space. However, between frames (∼3 ms) the diffusion becomes constrained by the tether, and the particle is likely to appear at a different location within the bounds of its tethered motion. This change from frame-to-frame can be observed by finding localized high-frequency fluctuations in the interferometric signal corresponding to changes in PSF phase, position, and intensity. Thus, it was necessary to remove other high-frequency fluctuation information from the videos. This was done by treating the pixels of each FOV as a vector that changed over time and finding the principal components of the vector, representing fluctuations that affected the FOV as a whole [Fig. 2(a)]. Next, pixel-wise calculations of frame-to-frame variation allowed for the generation of a clear contrast signal produced by tethered particles according to Eq. (1), where $x_{i}\cdots x_{N}$ represents the series of pixel values for the video time series and $\bar{x}$ represents the mean pixel value,

$$\sqrt{\frac{1}{N-1}\sum_{i=2}^{N}\frac{{\left(x_{i}-x_{i-1}\right)}^{2}}{\bar{x}}}.$$ (1)

**FIG. 3.**
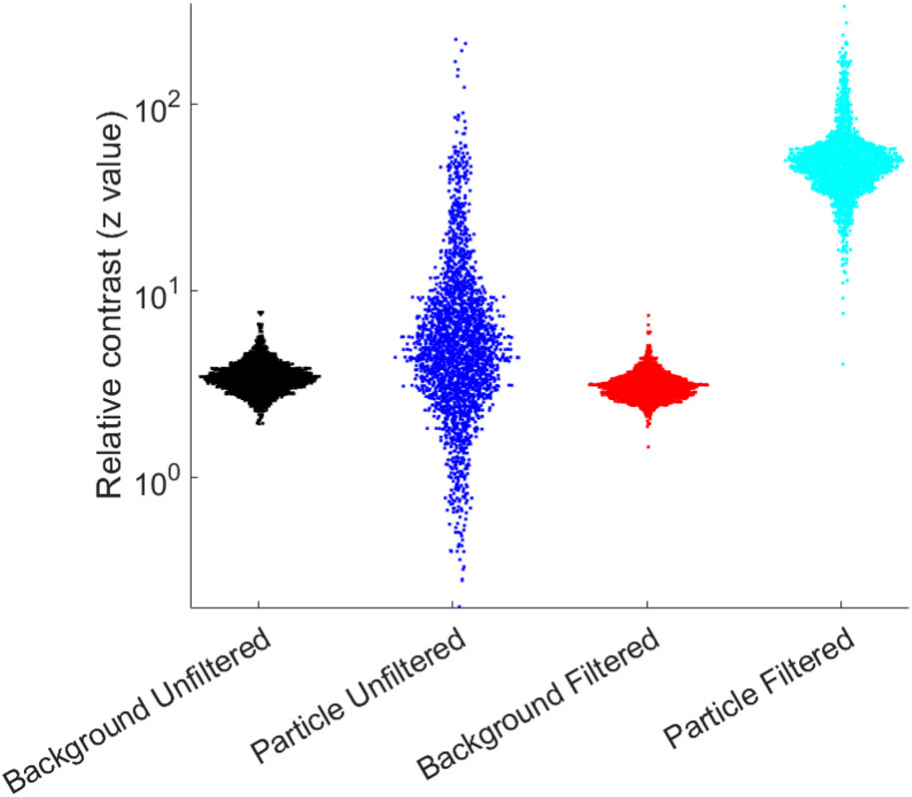
Change in contrast after image processing. For each FOV, a region of interest (ROI) containing no particle (background) and an ROI containing an immobilized pseudovirus (particle) were selected. Each ROI was subjected to a Gaussian smoothing kernel followed by the video analysis calculation in Eq. (1). For n = 2388 bound particles, the maximum signal within the particle ROI was compared to the signal of the background via a z-value calculation. For the FOV that was not subjected to PCA noise filtering, each particle's signal had a wide distribution that was not generally distinguishable from the signal in a background location. However, after PCA noise removal, the signal for each particle became clearly distinct from the background. The average contrast after PCA filtering was approximately z = 52, meaning that particles exhibited a signal that was greater than the background mean by 52 times the standard deviation of the background.

Figure 3 demonstrates the change in the contrast for particles as compared to the background of each video after filtering was applied. Without principal component analysis (PCA) filtering, the differential signals of each FOV were largely dominated by other sources of noise that were more significant than the particle fluctuations. However, by removing rapidly changing signals that affected the FOV as a whole, only the movements of particles remained, which are more clearly distinguished from background contributions.

**FIG. 4.**
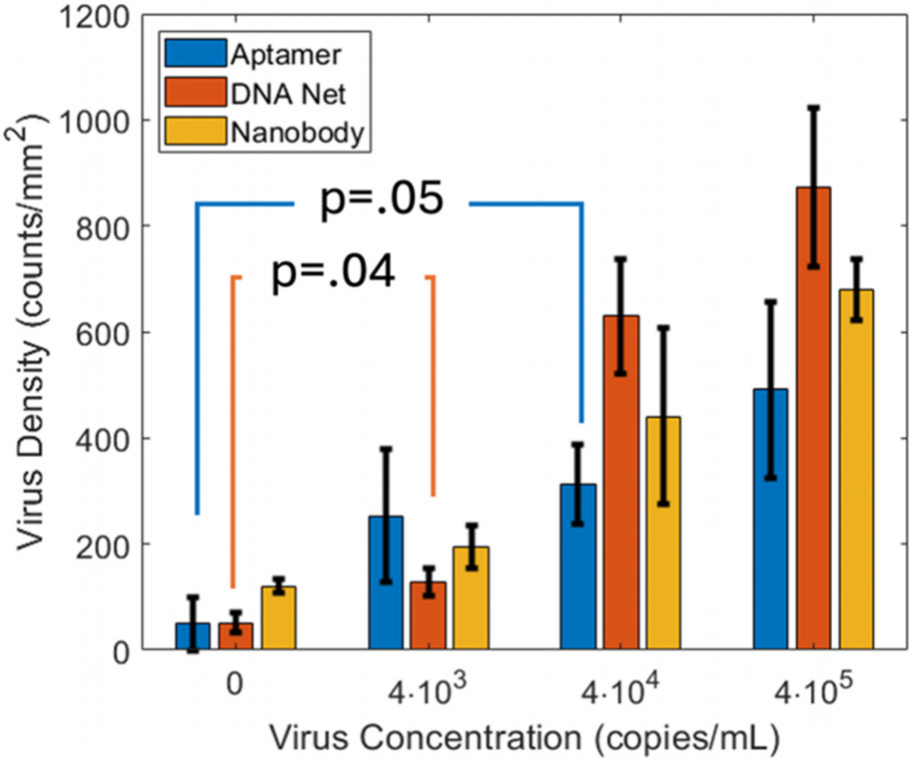
Dose–response characterization of viruses counted on the surface at different concentrations when captured using each molecule. An HIV pseudovirus from a stock of 4 × 10^5^ copies/ml was serially diluted and incubated on the functionalized surface for 30 min; for the blank measurement, buffer without virus was incubated on the surface for the same period of time. For all three capture molecules, the limit of detection (LOD) determined by 3× the standard deviation of the blank measurement results in an LOD between 4000 and 40 000 copies/ml. Each experimental group had independent samples N ≥ 4 for each trial; error bars represent standard error of the mean (SEM) or the mean of each population divided by √N. Significance was calculated using a two-sample t test.

The ability to examine multiple capturing strategies was made possible by the modular tethering strategy: each molecule had the same ssDNA tether of 20 bases (approximately 10 nm), which was complementary to a strand bound to the surface. Thus, the surface chemistry and preparation steps were identical in each experimental condition until the capture molecule was added. Another difference between this study and our previous work was an emphasis on efficiency and adaptation to the point-of-care environment. Reducing the incubation time of the test sample to 30 min creates the potential for sample-to-answer in less than an hour. An improved image tiling and autofocus code also allowed imaging to be completed in less than 15 min. For viral loads above 10^4^ copies/ml, or about 160 virions/sample, this strategy detects viruses rapidly; as seen in Fig. 4, the 4 × 10^4^ copies/ml sample was clearly differentiated from the negative sample. However, the lower concentrations were not distinguishable, assuming the requirement for a threshold for positive identification three standard deviations greater than the blank density. The DDNs captured a statistically significant number of particles at 4 × 10^3^ copies/ml, which approaches the typical limit of detection for laboratory-based PCR assays. Detection of virions is limited by diffusion, as they must come in contact with the sensor at the bottom of the 40 *μ*l chamber (see the supplementary material for analysis).

**FIG. 5.**
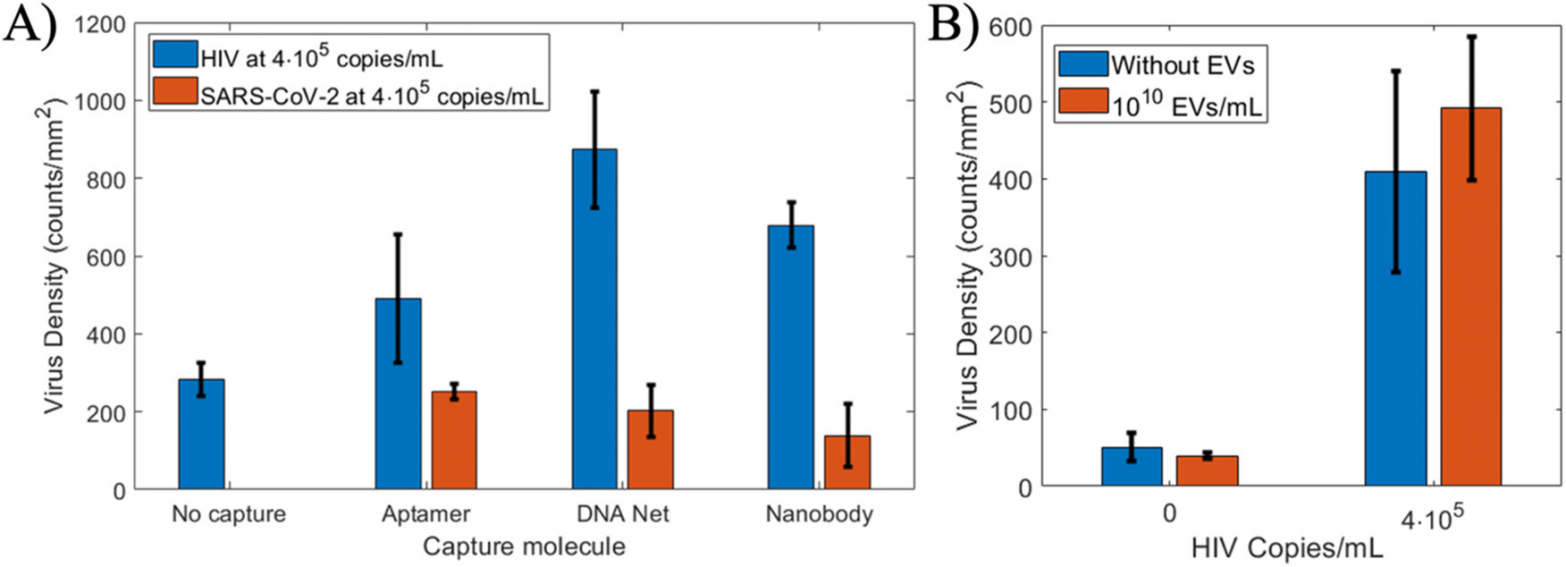
Selectivity of capture. (a) Analysis of nonspecific binding of HIV and selectivity of capture molecules against SARS-CoV-2. A surface with the same blocking treatment and viral concentration but no capture molecules was prepared to determine the capture rate of viruses by the undecorated surface. This result indicates that better blocking for the surface is necessary to prevent nonspecific binding. The SARS-CoV-2 comparison shows that the net and nanobody have, in general, more favorable selectivity than the aptamer. N = 3 for all SARS-CoV-2 data, and error bars represent SEM. (b) Selectivity against extracellular vesicles. Density of particles bound to DDNs when viral samples were combined with high-concentration extracellular vesicles (EVs) derived from U-87 MG cell culture. The blank trial indicates that very few EVs bind to the surface nonspecifically.

We sought to characterize the selectivity of the capture molecules to demonstrate the extent of nonspecific binding of a non-target virus and extracellular vesicle (EV) nanoparticles commonly found in clinical samples that are similar in size to HIV but lack gp120 outer proteins. Figure 5(a) shows the selectivity of the capture molecules for two viruses (HIV against SARS-CoV-2) and the nonspecific binding on the surface for viruses at the highest concentration (4 × 10^5^ copies/ml).

EVs in blood are present at concentrations that would typically overwhelm the population of viruses, and they are comparable in size. To test the feasibility of using this system with clinical samples, EVs derived from cell culture were spiked into blank and high-viral load dilutions and incubated on the surface decorated with DDNs. Consistent with typical measurements of EV concentrations in blood,^47^ a dilution of 10^10^ EV/ml was used, which means the viruses were outnumbered by four orders of magnitude. Even with these overwhelming numbers, the inclusion of an EV population did not significantly change the number of background particles captured in the blank sample. While the nonspecific binding from other virus populations remains a challenge for this method, non-virus particles that are similar in size did not bind to the immobilized DDNs [Fig. 5(b)]. The detection limit of our approach is dependent upon the number of nonspecific binding events present in baseline measurements, which we observe as non-zero. As our experiments are performed in an ordinary lab environment, there is potential for non-virus particulate matter to attach to the surface during the functionalization steps or during the assay. The future application of this assay for the study of clinical samples will depend on effective discrimination between viruses and other small particles present in plasma, so improved sample filtration and computational techniques for this task are topics of ongoing studies conducted by our group and collaborators. One avenue for the improvement of surface passivity was the application of a PEG blocking layer according to previously published protocols.^71^ This may be done on the TiO_2_ surface, or the design of the photonic crystal may be altered slightly to accommodate a SiO_2_ top layer that maintains the same optical properties. In either case, the PEG layer has the benefit of preventing some nonspecific binding but maintaining the modularity of the surface, since the same DNA-tether strategy may be used to attach any affinity molecule. The only modification is to use a biotin-functionalized ssDNA rather than amine-functionalized. Further surface optimizations will represent a trade-off between the sensitivity and complexity of the method and are the subject of future research.

## CONCLUSIONS

PRISM is an imaging platform for label-free particle detection that was leveraged in this study for the detection of intact HIV virions. Through the use of a modular single-stranded DNA tethering system, three different capturing molecules were evaluated to determine their effectiveness and affinity. This work also highlights an image processing technique that allowed the detection and digital counting of virions by differentiating between tethered particles and other sources of pixel variation. Analyzing the dose–response results, we determined that the 30-min incubation used in this assay was sufficient to distinguish the presence of virus at a concentration of 4 × 10^3^ copies/ml when captured by DDNs in buffer. The other two methods of capture, aptamer and nanobody, showed a higher limit of detection for this assay, but all three succeeded in immobilizing viruses such that PRISM could distinguish their scattering contributions from the background with a signal-to-noise ratio in excess of 50. The system was also robust to the presence of extracellular vesicles at high concentrations, giving an indication of the platform's ability to reject non-immobilized biological particles from detection. The specific isolation of tethered viruses by their constrained diffusion behavior on the photonic crystal surface is a promising result for future label-free particle counting studies, and PRISM has proven its versatility as a platform for the design and testing of diagnostic technologies.

## METHODS

### Materials

PP1-ZPUA UV-curable polymer and acrylsilane were purchased from Gelest. Pierce Protein-Free Blocking Buffer (TBS) was purchased from Thermo Fisher. PDMS Sylgard 184 kits were purchased from Krayden and Fisher Scientific. (3-Glycidyloxypropyl)trimethoxysilane (GPTMS) was purchased from Millipore Sigma and stored in a vacuum desiccator after opening for no more than a month to prevent decomposition. Repel-silane was purchased from Millipore Sigma. All oligonucleotides were purchased from Integrated DNA Technologies. 4-Methoxyphenyl 2-azidoacetate was purchased from Iris Biotech GmbH. The nanobody protein was bought from Bon Opus Biosciences. U-87 MG extracellular vesicles (EVs) were obtained from the lab of Professor Hisey.

### Extracellular vesicle production and isolation

U-87 MG EVs were produced using CELLine AD 1000 bioreactor flasks (Cole-Parmer Argos Technologies) as previously described.^67,68^ Briefly, 25 × 10^6^ U-87 MG cells were inoculated into the lower cell chamber of the bioreactor flask in complete Dulbecco's Modified Eagle Medium (DMEM, Gibco) with fetal bovine serum (FBS) and penicillin–streptomycin (PS), and roughly 750 ml of the same media was loaded into the upper media chamber. Over several weeks, the media in both chambers were adapted to DMEM/F-12 supplemented with chemically defined serum replacement CDM-HD (FiberCell Systems) and PS. The upper media chamber was refreshed once per week, and around 15 ml of conditioned media was harvested from the lower cell chamber thrice per week for roughly 4 months, with cell chamber media refreshed immediately following collection. EVs from the conditioned media were isolated using differential ultracentrifugation and size-exclusion chromatography (SEC). Suspended cells were first pelleted by centrifugation at 200 × g for 5 min, and debris was then pelleted by centrifugation at 2000 × g for 10 min. Crude large and small EVs were then isolated by differential ultracentrifugation by first pelleting large EVs at 20 000 × g for 30 min, followed by pelleting crude small EVs at 100 000 × g for 60 min and resuspending the pellet in 500 *μ*l phosphate-buffered saline (PBS). The small EVs were then further purified from co-isolated contaminants by collecting and pooling EV-rich fractions 1–4 from a 35 nm qEV Original SEC column (Izon Science). These U-87 MG small EVs were then used in the study.

### PC biosensor fabrication

PCs were fabricated using a slight modification of the method reported previously.^12^ Briefly, a silicon mold of a line-and-space pattern with 120 nm depth, 400 nm pitch, and 50% duty cycle was created using electron-beam lithography. This was cleaned with oxygen plasma descum and coated with repel-silane (Cytiva) to prevent adhesion. Glass coverslips were degreased with solvent (Acetone, IPA, and milliQ water), treated by oxygen plasma for 10 min (1 mTorr pressure, 200 W), and immersed in a solution of 5% acrylsilane in 20:1 EtOH:H_2_O adjusted to pH 4 for 5 min. Slides were rinsed in EtOH, blown with a nitrogen stream, and baked for 3 min at 110 °C. PP1-ZPUA UV-curable polymer was drop-cast onto the mold and covered by the treated coverslip; this was cured under a xenon lamp for 40 s before being peeled off of the mold and repeated on another coverslip. A Lesker dielectric sputterer was used to add approximately 96 nm of TiO_2_ to this surface (verified by ellipsometer reading) to achieve a resonant wavelength of 630 ± 3 nm.

### PC surface functionalization

As described previously,^12^ PCs were degreased and treated with oxygen plasma before being placed in a vacuum oven at 80 °C for 6 h in a chamber with liquid GPTMS (Millipore Sigma). The PCs were rinsed with toluene, methanol, and milliQ water. The tether strand (an oligonucleotide with an amine modification) was added to the PC surface at 50 *μ*M in a borate buffer with pH 8.5. These were equilibrated overnight at 4 °C.

### DDN and nanobody conjugation

We employed DDN design principles to create a 4 × 4 DNA Net (60 × 60 nm^2^), consisting of four rhombus units in both the x and y directions, with each rhombus having edges approximately 15 nm in length as detailed in Chauhan *et al.*^57^ The DNA Net was designed to position gp120 spike-targeting aptamers into a complex array of tri-aptamer clusters, featuring intra-tri-aptamer spacing of approximately 6 nm and inter-tri-aptamer spacing of approximately 15 nm, which matches the spacing of spike trimers on viral particles.^69^ The conjugation of DNA with nanobody was processed as previously reported.^70^ The nanobody was first reacted with 120-fold of 4-methoxyphenyl 2-azidoacetate for 3 days. Before the addition of DBCO modified DNA, the nanobody was washed using a 3 kDa Amicon centrifugal filter. The nanobody and DNA reaction was kept in 1× PBS buffer for 24 h. Conjugated nanobodies were stored at −20 °C.

### Sample preparation

Aptamer solutions were dissolved in STAR buffer (40 mM Tris-acetate, 2 mM EDTA, 12.5 mM magnesium chloride, 10 mM potassium chloride, pH 7.5, passed through a sterile 0.22 *μ*m syringe filter) to a concentration of 1 *μ*M and heated to 90 °C to ensure proper folding before application to the slide. DNA nanostructures and nanobody–DNA conjugates were diluted to 10 nM in the same buffer. After incubation on the sample for 30 min, unbound capture molecules were removed by gently washing the surface and replacing the buffer without allowing the surface to dry at any point. 50% TBS Pierce Protein-Free Blocking Buffer (also filtered via a 0.22 *μ*m syringe filter) was added for 30 min, followed by a rinse with STAR buffer to remove excess blocking buffer. Serially diluted pseudovirus in STAR buffer supplemented with 10% MedSchenker Smart Transport Media was added to the slides in 40 *μ*l 4 mm diameter wells and placed on a shaker at 600 rpm for 30 min. The surface was rinsed twice with the same buffer the viruses were diluted in and sealed with a glass coverslip. For EV spike-in trials, a sample of U-87 MG-derived EVs was evaluated using Nanoparticle Tracking Analysis (NTA) on a Malvern NanoSight NS300 instrument and determined to have an average diameter of 160 nm and a concentration of 5 × 10^12^ particles/ml; this was diluted by 500-fold and added to the virus solution before incubation. For EV trials, the volume of washing buffer was doubled to ensure removal of large particles.

### Imaging

Imaging occurred on the PRISM microscope previously described, shown in Fig. 1(a).^12,46^ Briefly, the PRISM optics are a simple array built on top of a commercial Zeiss Axio Observer 7 microscope base. A HeNe laser beam is collimated and directed through a half-wave plate to precisely control the polarization angle with respect to the stage. A doublet focuses this light onto the back focal plane of the illumination objective (Olympus LMPLFLN50x) resulting in Köhler illumination at the sample plane. A custom sample holder supports the PC above the imaging objective (Zeiss Plan-Apochromat 100×/1.46 Oil DIC), and the interference signal from the reference beam and scattered photons are directed to a CCD camera (FLIR GS3-U3-23S6M). Each well was imaged at 300 fps for 1.6 s/FOV with a shutter speed of 0.05 ms; FOVs are 512 × 512 pixels and approximately 30 × 30 *μ*m^2^. An automatic scanning and focus adjustment allowed at least 50 FOV to be efficiently captured for each independent well.

### Image processing

PRISM videos were processed to highlight regions of rapid, localized pixel variation independent of laser speckle; these diffraction-limited sources of fluctuation correspond to particles immobilized at the surface. First, laser speckle and mechanical vibrations were removed from the video by passing the frames' pixels as a vector into a principal component analysis (PCA) and subtracting the highest-intensity fluctuations to remove systematic variation that affected the entire video field [Fig. 2(a), see also supplementary material E]. The resulting videos were convolved with a small Gaussian kernel to smooth noise and highlight contributions from adjacent pixels that fluctuate together systematically. The videos were transformed into images: the time average of each pixel, the standard deviation of each pixel, and the RMS of frame-to-frame differences in each pixel [Fig. 2(b)]. The latter two frames were normalized by the square root of the intensity of the average pixel to remove the bias from total light intensity across the FOV. The summary images were displayed side-by-side, and regions of significant variation that did not correspond with a defect visible in the bright-field average image were selected [Fig. 2(b)]; these regions were consistent with the size of the expected point spread function (PSF) for immobilized particles on the PC surface. Bias was controlled for in the selection process by randomizing the order of analyzed videos and mixing videos from “positive” and “negative” samples together in a blinded procedure.

## SUPPLEMENTARY MATERIAL

See the supplementary material for additional information on methods and characterization. Please see the associated document for a more detailed mathematical description of the video processing, an example of a calibration of the sensor using nanoparticle binding, a description of the mass transport governing the binding of viruses to the photonic crystal surface, and information about DNA sequences used in the experiments.

## Data Availability

The data that support the findings of this study are available from the corresponding author upon reasonable request.

## References

[1] C. J. Achenbach, A. L. Buchanan, S. R. Cole, L. Hou, M. J. Mugavero, H. M. Crane, R. D. Moore, R. H. Haubrich, S. Gopal, J. J. Eron, P. W. Hunt, B. Rodriguez, K. Mayer, M. S. Saag, M. M. Kitahata, and Systems ftCfARNoIC. “HIV viremia and incidence of non-Hodgkin lymphoma in patients successfully treated with antiretroviral therapy,” Clin. Infect. Dis. 58(11), 1599–1606 (2014). 10.1093/cid/ciu076 24523217 PMC4017888

[2] S. D. Ramsey, J. M. Unger, L. H. Baker, R. F. Little, R. Loomba, J. P. Hwang, R. Chugh, M. A. Konerman, K. Arnold, and A. R. Menter, “Prevalence of hepatitis B virus, hepatitis C virus, and HIV infection among patients with newly diagnosed cancer from academic and community oncology practices,” JAMA Oncol. 5(4), 497–505 (2019). 10.1001/jamaoncol.2018.6437 30653226 PMC6459217

[3] Centers for Disease Control and Prevention, *Estimated HIV Incidence and Prevalence in the United States, 2018–2022* (U.S. Department of Health and Human Services, 2024), https://www.cdc.gov/hiv-data/nhss/estimated-hiv-incidence-and-prevalence.html.

[4] U.S. Department of Health and Human Services, *Viral Hepatitis National Strategic Plan for the United States: A Roadmap to Elimination (2021–2025)* (Department of Health and Human Services, Washington, DC, 2020).

[5] R. W. Peeling, D. I. Boeras, F. Marinucci, and P. Easterbrook, “The future of viral hepatitis testing: Innovations in testing technologies and approaches,” BMC Infect. Dis. 17(1), 187–196 (2017). 10.1186/s12879-017-2775-0 29143676 PMC5688478

[6] P. K. Drain, J. Dorward, A. Bender, L. Lillis, F. Marinucci, J. Sacks, A. Bershteyn, D. S. Boyle, J. D. Posner, and N. Garrett, “Point-of-care HIV viral load testing: An essential tool for a sustainable global HIV/AIDS response,” Clin. Microbiol. Rev. 32(3), e00097-18 (2019). 10.1128/cmr.00097-18 31092508 PMC6589862

[7] E. A. Ochodo, E. E. Olwanda, J. J. Deeks, and S. Mallett, “Point-of-care viral load tests to detect high HIV viral load in people living with HIV/AIDS attending health facilities,” Cochrane Database Syst. Rev. 3(3), CD013208 (2022). 10.1002/14651858.CD013208.pub2 35266555 PMC8908762

[8] A. Bacon, W. Wang, H. Lee, S. Umrao, P. D. Sinawang, D. Akin, K. Khemtonglang, A. Tan, S. Hirshfield, U. Demirci, X. Wang, and B. T. Cunningham, “Review of HIV self testing technologies and promising approaches for the next generation,” Biosensors 13(2), 298 (2023). 10.3390/bios13020298 36832064 PMC9954708

[9] N. Beard and A. Hill, “Combined “test and treat” campaigns for human immunodeficiency virus, hepatitis B, and hepatitis C: A systematic review to provide evidence to support World Health Organization treatment guidelines,” Open Forum Infect. Dis. 11(2), ofad666 (2024). 10.1093/ofid/ofad666 38352158 PMC10863549

[10] S. Wang, F. Xu, and U. Demirci, “Advances in developing HIV-1 viral load assays for resource-limited settings,” Biotechnol. Adv. 28(6), 770–781 (2010). 10.1016/j.biotechadv.2010.06.004 20600784 PMC2946488

[11] J. Zhao, L. Chang, and L. Wang, “Nucleic acid testing and molecular characterization of HIV infections,” Eur. J. Clin. Microbiol. Infect. Dis. 38(5), 829–842 (2019). 10.1007/s10096-019-03515-0 30798399

[12] N. Li, X. Wang, J. Tibbs, C. Che, A. S. Peinetti, B. Zhao, L. Liu, P. Barya, L. Cooper, L. Rong, X. Wang, Y. Lu, and B. T. Cunningham, “Label-free digital detection of intact virions by enhanced scattering microscopy,” J. Am. Chem. Soc. 144(4), 1498–1502 (2022). 10.1021/jacs.1c09579 34928591 PMC9762554

[13] L. Liu, J. Tibbs, N. Li, A. Bacon, S. Shepherd, H. Lee, N. Chauhan, U. Demirci, X. Wang, and B. T. Cunningham, “A photonic resonator interferometric scattering microscope for label-free detection of nanometer-scale objects with digital precision in point-of-use environments,” Biosens. Bioelectron. 228, 115197 (2023). 10.1016/j.bios.2023.115197 36905862 PMC10072782

[14] R. Ahmed, C. F. Guimarães, J. Wang, F. Soto, A. H. Karim, Z. Zhang, R. L. Reis, D. Akin, R. Paulmurugan, and U. Demirci, “Large-scale functionalized metasurface-based SARS-CoV-2 detection and quantification,” ACS Nano 16(10), 15946–15958 (2022). 10.1021/acsnano.2c02500 36125414

[15] F. Inci, O. Tokel, S. Wang, U. A. Gurkan, S. Tasoglu, D. R. Kuritzkes, and U. Demirci, “Nanoplasmonic quantitative detection of intact viruses from unprocessed whole blood,” ACS Nano 7(6), 4733–4745 (2013). 10.1021/nn3036232 23688050 PMC3700402

[16] V. Shpacovitch, V. Temchura, M. Matrosovich, J. Hamacher, J. Skolnik, P. Libuschewski, D. Siedhoff, F. Weichert, P. Marwedel, and H. Müller, “Application of surface plasmon resonance imaging technique for the detection of single spherical biological submicrometer particles,” Anal. Biochem. 486, 62–69 (2015). 10.1016/j.ab.2015.06.022 26095398

[17] Y. Jiang, C. Y. Tan, S. Y. Tan, M. S. F. Wong, Y. F. Chen, L. Zhang, K. Yao, S. K. E. Gan, C. Verma, and Y.-J. Tan, “SAW sensor for Influenza A virus detection enabled with efficient surface functionalization,” Sens. Actuators, B 209, 78–84 (2015). 10.1016/j.snb.2014.11.103

[18] C. Cheng, H. Cui, J. Wu, and S. Eda, “A PCR-free point-of-care capacitive immunoassay for influenza A virus,” Microchim. Acta 184, 1649–1657 (2017). 10.1007/s00604-017-2140-4

[19] P. Gorelkin, A. Erofeev, G. Kiselev, D. Kolesov, E. Dubrovin, and I. Yaminsky, “Synthetic sialylglycopolymer receptor for virus detection using cantilever-based sensors,” Analyst 140(17), 6131–6137 (2015). 10.1039/C5AN01102G 26215598

[20] Z. Zhang, P. Ma, R. Ahmed, J. Wang, D. Akin, F. Soto, B. F. Liu, P. Li, and U. Demirci, “Advanced point-of-care testing technologies for human acute respiratory virus detection,” Adv. Mater. 34(1), e2103646 (2022). 10.1002/adma.202103646 34623709

[21] J. Lee, S. R. Ahmed, S. Oh, J. Kim, T. Suzuki, K. Parmar, S. S. Park, J. Lee, and E. Y. Park, “A plasmon-assisted fluoro-immunoassay using gold nanoparticle-decorated carbon nanotubes for monitoring the influenza virus,” Biosens. Bioelectron. 64, 311–317 (2015). 10.1016/j.bios.2014.09.021 25240957

[22] L. F. Yang, N. Kacherovsky, N. Panpradist, R. Wan, J. Liang, B. Zhang, S. J. Salipante, B. R. Lutz, and S. H. Pun, “Aptamer sandwich lateral flow assay (AptaFlow) for antibody-free SARS-CoV-2 detection,” Anal. Chem. 94(20), 7278–7285 (2022). 10.1021/acs.analchem.2c00554 35532905 PMC9112978

[23] L.-D. Xu, F.-L. Du, J. Zhu, and S.-N. Ding, “Luminous silica colloids with carbon dot incorporation for sensitive immunochromatographic assay of Zika virus,” Analyst 146(2), 706–713 (2021). 10.1039/D0AN02017F 33216074

[24] C. Wang, C. Wang, X. Wang, K. Wang, Y. Zhu, Z. Rong, W. Wang, R. Xiao, and S. Wang, “Magnetic SERS strip for sensitive and simultaneous detection of respiratory viruses,” ACS Appl. Mater. Interfaces 11(21), 19495–19505 (2019). 10.1021/acsami.9b03920 31058488

[25] N. Wiriyachaiporn, S. Sirikaew, S. Bamrungsap, T. Limcharoen, P. Polkankosit, P. Roeksrungruang, and K. Ponlamuangdee, “A simple fluorescence-based lateral flow test platform for rapid influenza B virus screening,” Anal. Methods 13(14), 1687–1694 (2021). 10.1039/D0AY01988G 33861235

[26] T. Rozmyslowicz, J. deSa, R. Lec, and G. N. Gaulton, “A novel point-of-care BioNanoSensor for rapid HIV detection and treatment monitoring,” J. AIDS Clin. Res. 6(5), 454 (2015). 10.4172/2155-6113.1000454 26457228 PMC4596080

[27] M. Bisoffi, V. Severns, D. W. Branch, T. L. Edwards, and R. S. Larson, “Rapid detection of human immunodeficiency virus types 1 and 2 by use of an improved piezoelectric biosensor,” J. Clin. Microbiol. 51(6), 1685–1691 (2013). 10.1128/JCM.03041-12 23515541 PMC3716090

[28] A. M. Shrivastav, U. Cvelbar, and I. Abdulhalim, “A comprehensive review on plasmonic-based biosensors used in viral diagnostics,” Commun. Biol. 4(1), 70 (2021). 10.1038/s42003-020-01615-8 33452375 PMC7810758

[29] H. Shafiee, E. A. Lidstone, M. Jahangir, F. Inci, E. Hanhauser, T. J. Henrich, D. R. Kuritzkes, B. T. Cunningham, and U. Demirci, “Nanostructured optical photonic crystal biosensor for HIV viral load measurement,” Sci. Rep. 4, 4116 (2014). 10.1038/srep04116 24576941 PMC3937800

[30] Y.-T. Chen, Y.-Y. Liao, C.-C. Chen, H.-H. Hsiao, and J.-J. Huang, “Surface plasmons coupled two-dimensional photonic crystal biosensors for Epstein-Barr virus protein detection,” Sens. Actuators, B 291, 81–88 (2019). 10.1016/j.snb.2019.04.059

[31] M. Z. H. Khan, M. R. Hasan, S. I. Hossain, M. S. Ahommed, and M. Daizy, “Ultrasensitive detection of pathogenic viruses with electrochemical biosensor: State of the art,” Biosens. Bioelectron. 166, 112431 (2020). 10.1016/j.bios.2020.112431 32862842 PMC7363606

[32] G. Young and P. Kukura, “Interferometric scattering microscopy,” Annu. Rev. Phys. Chem. 70, 301–322 (2019). 10.1146/annurev-physchem-050317-021247 30978297

[33] R. W. Taylor and V. Sandoghdar, “Interferometric scattering microscopy: Seeing single nanoparticles and molecules via Rayleigh scattering,” Nano Lett. 19(8), 4827–4835 (2019). 10.1021/acs.nanolett.9b01822 31314539 PMC6750867

[34] M. Piliarik and V. Sandoghdar, “Direct optical sensing of single unlabelled proteins and super-resolution imaging of their binding sites,” Nat. Commun. 5, 4495 (2014). 10.1038/ncomms5495 25072241

[35] A. Y. Ozkumur, F. E. Kanik, J. T. Trueb, C. Yurdakul, and M. S. Unlu, “Interferometric detection and enumeration of viral particles using Si-based microfluidics,” IEEE J. Sel. Top. Quantum Electron. 25(1), 1–8 (2019). 10.1109/JSTQE.2018.2854548

[36] P. Kukura, H. Ewers, C. Muller, A. Renn, A. Helenius, and V. Sandoghdar, “High-speed nanoscopic tracking of the position and orientation of a single virus,” Nat. Methods 6(12), 923–927 (2009). 10.1038/nmeth.1395 19881510

[37] H. M. Dastjerdi, R. G. Mahmoodabadi, M. Bär, V. Sandoghdar, and H. Köstler, “PiSCAT: A Python package for interferometric scattering microscopy,” J. Open Source Software 7(71), 4024 (2022). 10.21105/joss.04024

[38] G. G. Daaboul, C. A. Lopez, J. Chinnala, B. B. Goldberg, J. H. Connor, and M. S. Unlu, “Digital sensing and sizing of vesicular stomatitis virus pseudotypes in complex media: A model for Ebola and Marburg detection,” ACS Nano 8(6), 6047–6055 (2014). 10.1021/nn501312q 24840765 PMC4466106

[39] G. Young, N. Hundt, D. Cole, A. Fineberg, J. Andrecka, A. Tyler, A. Olerinyova, A. Ansari, E. G. Marklund, M. P. Collier, S. A. Chandler, O. Tkachenko, J. Allen, M. Crispin, N. Billington, Y. Takagi, J. R. Sellers, C. Eichmann, P. Selenko, L. Frey, R. Riek, M. R. Galpin, W. B. Struwe, J. L. P. Benesch, and P. Kukura, “Quantitative mass imaging of single biological macromolecules,” Science 360(6387), 423–427 (2018). 10.1126/science.aar5839 29700264 PMC6103225

[40] J. Ortega Arroyo, J. Andrecka, K. M. Spillane, N. Billington, Y. Takagi, J. R. Sellers, and P. Kukura, “Label-free, all-optical detection, imaging, and tracking of a single protein,” Nano Lett. 14(4), 2065–2070 (2014). 10.1021/nl500234t 24597479 PMC4186656

[41] G. De Angelis, J. Abramo, M. Miasnikova, M. Taubert, C. Eggeling, and F. Reina, “Homogeneous large field-of-view and compact iSCAT-TIRF setup for dynamic single molecule measurements,” Opt. Express 32(26), 46607 (2024). 10.1364/oe.532947

[42] T. Heermann, F. Steiert, B. Ramm, N. Hundt, and P. Schwille, “Mass-sensitive particle tracking to elucidate the membrane-associated MinDE reaction cycle,” Nat. Methods 18(10), 1239–1246 (2021). 10.1038/s41592-021-01260-x 34608318 PMC8490154

[43] C. Y. Cheng, Y. H. Liao, and C. L. Hsieh, “Dynamic signal of live biological cells under interferometric scattering (iSCAT) microscopy and its impacts on single-particle tracking,” J. Phys. D 54(36), 364001 (2021). 10.1088/1361-6463/ac083e

[44] G. Ma, Z. Wan, Y. Yang, P. Zhang, S. Wang, and N. Tao, “Optical imaging of single-protein size, charge, mobility, and binding,” Nat. Commun. 11(1), 4768 (2020). 10.1038/s41467-020-18547-w 32958747 PMC7505846

[45] C. Yurdakul, O. Avci, A. Matlock, A. J. Devaux, M. V. Quintero, E. Ozbay, R. A. Davey, J. H. Connor, W. C. Karl, L. Tian, and M. S. Unlu, “High-throughput, high-resolution interferometric light microscopy of biological nanoparticles,” ACS Nano 14(2), 2002–2013 (2020).10.1021/acsnano.9b0851232003974

[46] N. Li, T. D. Canady, Q. Huang, X. Wang, G. A. Fried, and B. T. Cunningham, “Photonic resonator interferometric scattering microscopy,” Nat. Commun. 12(1), 1744 (2021). 10.1038/s41467-021-21999-3 33741998 PMC7979857

[47] K. B. Johnsen, J. M. Gudbergsson, T. L. Andresen, and J. B. Simonsen, “What is the blood concentration of extracellular vesicles? Implications for the use of extracellular vesicles as blood-borne biomarkers of cancer,” Biochim. Biophys. Acta 1871(1), 109–116 (2019). 10.1016/j.bbcan.2018.11.006 30528756

[48] S. M. Scherr, G. G. Daaboul, J. Trueb, D. Sevenler, H. Fawcett, B. Goldberg, J. H. Connor, and M. S. Unlu, “Real-time capture and visualization of individual viruses in complex media,” ACS Nano 10(2), 2827–2833 (2016). 10.1021/acsnano.5b07948 26760677 PMC5019356

[49] S. Wang, M. Esfahani, U. A. Gurkan, F. Inci, D. R. Kuritzkes, and U. Demirci, “Efficient on-chip isolation of HIV subtypes,” Lab Chip 12(8), 1508–1515 (2012). 10.1039/c2lc20706k 22391989 PMC3777392

[50] S. Nandi, A. Mondal, A. Roberts, and S. Gandhi, “Biosensor platforms for rapid HIV detection,” Adv. Clin. Chem. 98, 1–34 (2020). 10.1016/bs.acc.2020.02.001 32564784

[51] E. Seymour, M. S. Unlu, and J. H. Connor, “A high-throughput single-particle imaging platform for antibody characterization and a novel competition assay for therapeutic antibodies,” Sci. Rep. 13(1), 306 (2023). 10.1038/s41598-022-27281-w 36609657 PMC9821353

[52] A. D. Ellington and J. W. Szostak, “In vitro selection of RNA molecules that bind specific ligands,” Nature 346(6287), 818–822 (1990). 10.1038/346818a0 1697402

[53] C. K. O'Sullivan, “Aptasensors – The future of biosensing?,” Anal. Bioanal. Chem. 372(1), 44–48 (2002). 10.1007/s00216-001-1189-3 11939212

[54] B. Strehlitz, N. Nikolaus, and R. Stoltenburg, “Protein detection with aptamer biosensors,” Sensors 8(7), 4296–4307 (2008). 10.3390/s8074296 27879936 PMC3697175

[55] C. Lin, Y. Liu, S. Rinker, and H. Yan, “DNA tile based self-assembly: Building complex nanoarchitectures,” ChemPhysChem 7(8), 1641–1647 (2006). 10.1002/cphc.200600260 16832805

[56] P. S. Kwon, S. Ren, S. J. Kwon, M. E. Kizer, L. Kuo, M. Xie, D. Zhu, F. Zhou, F. Zhang, D. Kim, K. Fraser, L. D. Kramer, N. C. Seeman, J. S. Dordick, R. J. Linhardt, J. Chao, and X. Wang, “Designer DNA architecture offers precise and multivalent spatial pattern-recognition for viral sensing and inhibition,” Nat. Chem. 12(1), 26–35 (2020). 10.1038/s41557-019-0369-8 31767992 PMC6925649

[57] N. Chauhan, Y. Xiong, S. Ren, A. Dwivedy, N. Magazine, L. Zhou, X. Jin, T. Zhang, B. T. Cunningham, S. Yao, W. Huang, and X. Wang, “Net-shaped DNA nanostructures designed for rapid/sensitive detection and potential inhibition of the SARS-CoV-2 virus,” J. Am. Chem. Soc. 145(37), 20214 (2023). 10.1021/jacs.2c04835 35881910 PMC9344894

[58] H. Lee, W. Wang, N. Chauhan, Y. Xiong, N. Magazine, O. Valdescruz, D. Y. Kim, T. Qiu, W. Huang, and X. Wang, “Rapid detection of intact SARS-CoV-2 using designer DNA Nets and a pocket-size smartphone-linked fluorimeter,” Biosens. Bioelectron. 229, 115228 (2023). 10.1016/j.bios.2023.115228 36963325 PMC10019040

[59] N. Li, B. Zhao, R. Stavins, A. S. Peinetti, N. Chauhan, R. Bashir, B. T. Cunningham, W. P. King, Y. Lu, X. Wang, and E. Valera, “Overcoming the limitations of COVID-19 diagnostics with nanostructures, nucleic acid engineering, and additive manufacturing,” Curr. Opin. Solid State Mater. Sci. 26(1), 100966 (2022). 10.1016/j.cossms.2021.100966 34840515 PMC8604633

[60] S. Umrao, M. Zheng, X. Jin, S. Yao, and X. Wang, “Net-shaped DNA nanostructure-based lateral flow assays for rapid and sensitive SARS-CoV-2 detection,” Anal. Chem. 96(8), 3291–3299 (2024). 10.1021/acs.analchem.3c03698 38306661 PMC10922791

[61] R. A. Weiss and C. T. Verrips, “Nanobodies that neutralize HIV,” Vaccines 7(3), 77 (2019). 10.3390/vaccines7030077 31370301 PMC6789485

[62] E. R. Gray, J. C. Brookes, C. Caillat, V. Turbe, B. L. J. Webb, L. A. Granger, B. S. Miller, L. E. McCoy, M. El Khattabi, C. T. Verrips, R. A. Weiss, D. M. Duffy, W. Weissenhorn, and R. A. McKendry, “Unravelling the molecular basis of high affinity nanobodies against HIV p24: In vitro functional, structural, and in silico insights,” ACS Infect. Dis. 3(7), 479–491 (2017). 10.1021/acsinfecdis.6b00189 28591513

[63] B. Cunningham, B. Lin, J. Qiu, P. Li, J. Pepper, and B. Hugh, “A plastic colorimetric resonant optical biosensor for multiparallel detection of label-free biochemical interactions,” Sens. Actuators, B 85(3), 219–226 (2002). 10.1016/S0925-4005(02)00111-9

[64] A. D. Boardman, D. P. Tsai, M. C. George, J. N. Liu, A. Farhang, B. Williamson, M. Black, T. Wangensteen, J. Fraser, R. Petrova, and B. T. Cunningham, “Wafer-scale plasmonic and photonic crystal sensors,” Proc. SPIE 9547, 9547iF (2015). 10.1117/12.2188631

[65] L. Liu, T. Ayupova, S. Umrao, L. D. Akin, H. K. Lee, J. Tibbs, X. Wang, U. Demirci, and B. T. Cunningham, “A biosensor-integrated filtration device for nanoparticle isolation and label-free imaging,” Lab Chip 25(8), 2073–2084 (2025). 10.1039/d5lc00089k 40105290 PMC11921766

[66] S. Umrao, A. Dwivedy, M. Zheng, V. Anirudhan, U. Parlatan, T. Henrich, B. T. Cunningham, U. Demirci, L. Rong, and X. Wang, “Broad-spectrum HIV-1 detection and neutralization via multivalent designer DNA nanostructures,” bioRxiv (2025). 10.1101/2025.09.21.677611

[67] A. Artuyants, V. Chang, G. Reshef, C. Blenkiron, L. W. Chamley, E. Leung, and C. L. Hisey, “Production of extracellular vesicles using a CELLine adherent bioreactor flask,” Methods Mol. Biol. 2436, 183–192 (2022). 10.1007/7651_2021_413 34490596

[68] C. L. Hisey, A. Artuyants, G. Guo, V. Chang, G. Reshef, M. Middleditch, B. Jacob, L. W. Chamley, and C. Blenkiron, “Investigating the consistency of extracellular vesicle production from breast cancer subtypes using CELLine adherent bioreactors,” J. Extracell. Biol. 1(9), e60 (2022). 10.1002/jex2.60 38938775 PMC11080891

[69] J. Liu, A. Bartesaghi, M. J. Borgnia, G. Sapiro, and S. Subramaniam, “Molecular architecture of native HIV-1 gp120 trimers,” Nature 455(7209), 109–113 (2008). 10.1038/nature07159 18668044 PMC2610422

[70] T. Song, L. Cooper, J. Galvan Achi, X. Wang, A. Dwivedy, L. Rong, and X. Wang, “Polyvalent nanobody structure designed for boosting SARS-CoV-2 inhibition,” J. Am. Chem. Soc. 146(9), 5894–5900 (2024). 10.1021/jacs.3c11760 38408177 PMC10965196

[71] T. Paul and S. Myong, “Protocol for generation and regeneration of PEG-passivated slides for single-molecule measurements,” STAR Protoc. 3(1), 101152 (2022). 10.1016/j.xpro.2022.101152 35146451 PMC8819390

